# Diagnostic Needle Arthroscopy of the Shoulder: A Validation Study

**DOI:** 10.1177/23259671231155885

**Published:** 2023-08-09

**Authors:** Alex Chowdhury, Catherine Gibson, Alex Nicholls, Iain MacLeod, Henry Colaco

**Affiliations:** * Hampshire Hospitals NHS Foundation Trust, Royal Hampshire County Hospital, Winchester, UK.; † John Radcliffe Hospital, Oxford, UK.; *Investigation performed at Royal Hampshire County Hospital, Winchester, UK*

**Keywords:** shoulder, general, imaging, magnetic resonance, clinical assessment/grading scales, arthroscopy

## Abstract

**Background:**

Diagnostic needle arthroscopy offers an alternative imaging modality to magnetic resonance imaging (MRI) for the diagnosis of intra-articular pathology.

**Purpose:**

To compare the accuracy of a needle arthroscopy device (Mi-eye2) versus MRI in identifying intra-articular anatomic abnormalities in the glenohumeral joint, with formal arthroscopy as the gold standard.

**Study Design:**

Cohort study; Level of evidence, 2.

**Methods:**

A total of 22 patients underwent diagnostic needle arthroscopy of the shoulder, of whom 20 had preoperative MRI scans. A standardized 12-point noninstrumented diagnostic arthroscopy was performed on each patient using the 0° needle arthroscope, followed by a 30°, 4 mm–diameter conventional arthroscope. Intraoperative images were randomized and reviewed by 2 independent blinded fellowship-trained shoulder surgeons for identification of key pathology and anatomic structures. The MRI scans were reviewed by a single musculoskeletal radiologist to identify pathology in the same key areas.

**Results:**

For the identification of rotator cuff pathology, needle arthroscopy (sensitivity, 0.75; specificity, 1.00) was superior to MRI (sensitivity, 0.75; specificity, 0.75) with an interobserver reliability (κ) of 0.703. For long head of the biceps pathology, needle arthroscopy (sensitivity, 0.67; specificity, 0.95) was superior to MRI (sensitivity, 0.00; specificity, 0.83). It was less accurate for labral (sensitivity, 0.33; specificity, 0.50; κ = 0.522) and articular cartilage pathology (sensitivity, 0.00; specificity, 0.94; κ = 0.353). The number of anatomic structures that could be clearly identified was 8.35 of 12 (69.58%) for needle arthroscopy versus 10.35 of 12 (86.25%) for standard arthroscopy.

**Conclusion:**

Diagnostic needle arthroscopy was found to be more accurate than MRI for the diagnosis of rotator cuff and long head of the biceps pathology but was less accurate for diagnosing labral and cartilage pathology. Although the field of view of a 0° needle arthroscope is not equivalent to a 30° conventional arthroscope, it presents an alternative with potential for use in an outpatient setting.

The development of in-office diagnostic needle arthroscopy has the potential to revolutionize the surgeon-patient interaction, permitting a point-of-contact comparison of clinical findings with joint visualization. Diagnostic arthroscopy is the gold standard for intra-articular assessment, allowing direct and dynamic visualization of anatomic structures. However, it is invasive, requires the use of general and/or regional anesthesia, and is time- and resource-consuming.^9^ Thus, it is most frequently performed after advanced imaging, with a therapeutic aim.

Magnetic resonance imaging (MRI) is a noninvasive advanced imaging modality that is of increasing use for conditions of the shoulder. Its accuracy is well established for a number of intra-articular pathologies, including full-thickness rotator cuff tears and labral lesions.^5,6^ However, it is contraindicated in some patients (eg, claustrophobia, implanted medical devices). Computed tomography arthrography offers an alternative but exposes patients to risks of ionizing radiation and contrast media. Furthermore, these are both static imaging modalities and are often associated with an increased waiting time (dependent on local health care provision).

Needle arthroscopy has been developed as a minimally invasive dynamic investigation that can be performed alongside initial clinical assessment. A number of studies have demonstrated the accuracy of such devices in the knee.^2,12^ There is only 1 previous report assessing the accuracy of this technology in the glenohumeral joint, revealing high specificities, although moderate sensitivities, for certain intra-articular pathologies, including full-thickness cuff tears and labral tears.^11^

The aim of this study was to assess the accuracy (including sensitivity, specificity, positive predictive value [PPV], and negative predictive value [NPV]) of needle arthroscopy in the glenohumeral joint, with the use of formal arthroscopy as the comparative gold standard. We hypothesized that needle arthroscopy would allow equivalent identification of intra-articular glenohumeral pathology (ie, anatomic abnormalities) when compared with MRI.

## Methods

### Patients

Ethics approval for the study protocol was waived by the local research governance department. Twenty-two patients (7 female, 15 male; age range, 16-84 years) were prospectively recruited over a 2-year period. All patients were scheduled to undergo therapeutic arthroscopy for a range of indications.

All 22 patients underwent needle arthroscopy, followed by formal arthroscopy with subsequent therapeutic intervention (eg, rotator cuff repair). Twenty of the patients had undergone 1.5-T MRI scanning before arthroscopy.

### Needle Arthroscopy Device

The Mi-eye2 system (Trice Medical) consists of a disposable handpiece with a light source and 0° camera, connected to a computer tablet device. The handpiece contains a sharp arthroscopy needle, with a 14-gauge outer sheath that is retractable upon joint entry, allowing for the deployment of the camera and light source. The attached Luer lock port can be connected to a syringe for injection of saline into the joint via the needle. Static images and video are recorded on the tablet and can be exported to a universal serial bus storage device.

### Diagnostic Procedure

Diagnostic needle arthroscopy was performed in the operating theater, before formal arthroscopy, by the senior author (H.C.). All patients were under general anesthesia and interscalene regional nerve block in a beach-chair position with in-line variable traction.

The sterile camera from the Mi-eye2 system was connected to the nonsterile tablet, placed outside the surgical field on a Mayo table in view of the operating surgeon. The needle arthroscope was introduced into the glenohumeral joint via a standard posterior viewing portal, after which the sharp entry sheath was retracted and sterile saline (30-60 mL) injected to improve safety and visualization.

**Figure 1.**
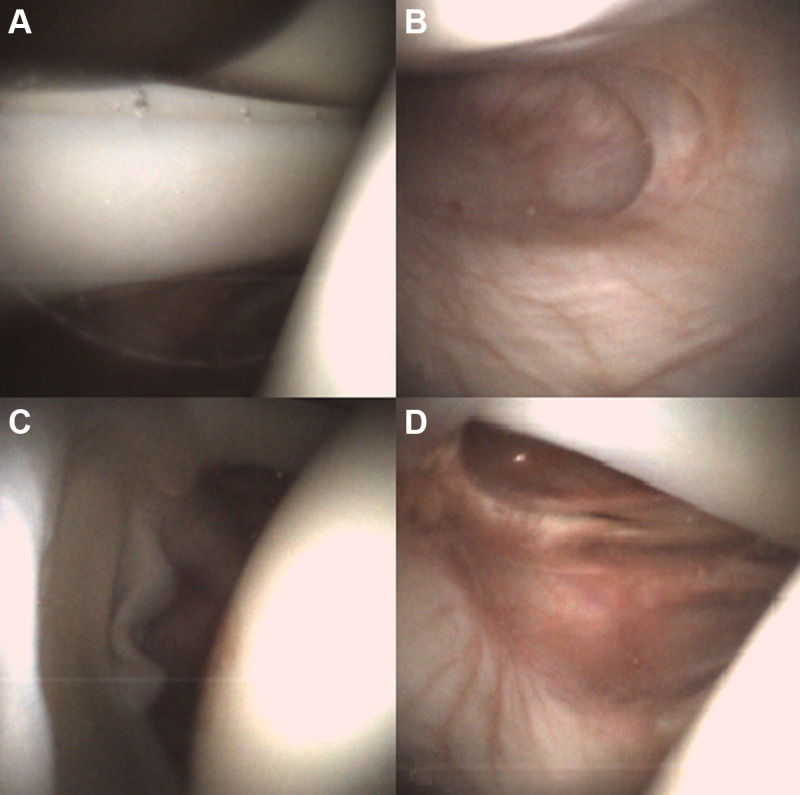
Glenohumeral joint images taken with the Mi-eye2 device: (A) long head of the biceps, (B) inferior recess, (C) posterior labrum, and (D) rotator interval (right shoulder, beach-chair position, posterior viewing portal).

Standardized 12-point diagnostic arthroscopy was performed on each patient, with static images recorded in each position (Figure 1). The positions were as follows: (1) biceps anchor, (2) long head of the biceps tendon, (3) long head of the biceps in the sheath/groove, (4) long head of the biceps pulley and subscapularis, (5) supraspinatus, (6) infraspinatus/bare area, (7) inferior recess, (8) posterior labrum, (9) glenoid, (10) humeral head, (11) anteroinferior labrum, and (12) rotator interval (including middle glenohumeral ligament).

The primary surgeon then proceeded to formal arthroscopy using a standard 30°, 4 mm–diameter conventional arthroscope. The same 12-point diagnostic routine was performed, with static images recorded of each anatomic landmark.

### Image Assessment

The image sets from both the Mi-eye2 device and formal arthroscopy were anonymized and randomized. They were then reviewed by 2 independent blinded fellowship-trained shoulder surgeons (I.M., A.N.). They were asked to input binary data (yes/no responses) for adequate visualization (identification of anatomy) of the 12 anatomic positions and for identification of pathology in 5 key areas (rotator cuff, labrum, long head of the biceps, articular cartilage, and rotator interval). The MRI scans were reviewed by a single musculoskeletal radiologist to identify pathology in the same key areas.

Pathology of the labrum was defined as a visible tear, displacement, or significant fraying. Pathology of the cartilage was defined as an Outerbridge grade >0. Pathology of the rotator cuff was defined as a full- or partial-thickness tear. Pathology of the long head of the biceps tendon was defined as a tear, fraying, or subluxation. Pathology of the rotator interval was defined as synovitis or thickening. Pathology of the middle glenohumeral ligament was defined as thickening and/or synovitis. The reviewers were also asked to suggest a diagnosis for each image set.

### Statistical Analysis

Statistical analysis was performed by an independent observer. Sensitivity, specificity, PPV, and NPV were calculated for the blinded reviewers’ Mi-eye2 image assessment, their formal arthroscopy image assessment, and the radiologist’s MRI assessment, using the intraoperative arthroscopic findings of the primary surgeon as the gold standard. The Cohen kappa coefficient (κ) was used to calculate intraobserver agreement between procedures (Mi-eye2 vs arthroscopy) and interobserver agreement with the Mi-eye2 (reviewer 1 vs 2), with the following levels of agreement: ≤0, none; 0.01 to 0.20, none to slight; 0.21 to 0.40, fair; 0.41 to 0.60, moderate; 0.61 to 0.80, substantial; and 0.81 to 1.00, almost perfect agreement.

## Results

Among the 22 study patients, there was 1 incidence of humeral head partial-thickness cartilage injury during needle arthroscope insertion, which did not require further intervention. There were no other complications after the diagnostic procedures.

### Visualization of Anatomic Positions

The blinded reviewers were able to satisfactorily identify 8.3 (69.2%) and 8.4 (70.0%) of the 12 standardized anatomic positions from the Mi-eye2 images. From the formal arthroscopy images, they were able to identify 10.2 (85%) and 10.5 (87.5%) of the positions. There were 3 positions (long head of the biceps anchor, long head of the biceps, and humeral head) that were adequately identified from the Mi-eye2 images in >80% of patients by each reviewer. There were also 3 positions (inferior recess, posterior labrum, and long head of the biceps in the sheath/groove) that were adequately visualized in <60% of patients by each reviewer (Table 1).

**Table 1:** Adequate Visualization by the Reviewers of Each Anatomic Position (Images From 22 Patients)*^a^*

	Formal Arthroscopy Images		Mi-eye2 Images	
Anatomic Position	Reviewer 1	Reviewer 2	Reviewer 1	Reviewer 2
1: LHB anchor	17 (77.3)	18 (81.8)	18 (81.8)	19 (86.4)
2: LHB	18 (81.8)	17 (77.3)	19 (86.4)	18 (81.8)
3: LHB in sheath/groove	19 (86.4)	17 (77.3)	13 (59.1)	13 (59.1)
4: LHB and pulley and SSc	21 (95.5)	18 (81.8)	14 (63.6)	13 (59.1)
5: SSp	20 (90.9)	19 (86.4)	17 (77.3)	17 (77.3)
6: ISp	19 (86.4)	21 (95.5)	13 (59.1)	14 (63.6)
7: Inferior recess	17 (77.3)	18 (81.8)	12 (54.5)	13 (59.1)
8: Posterior labrum	17 (77.3)	14 (63.6)	4 (18.2)	5 (22.7)
9: Glenoid	19 (86.4)	20 (90.9)	17 (77.3)	17 (77.3)
10: Humeral head	18 (81.8)	19 (86.4)	21 (95.5)	21 (95.5)
11: Anteroinferior labrum	19 (86.4)	16 (72.7)	18 (81.8)	17 (77.3)
12: Rotator interval	18 (81.8)	16 (72.7)	17 (77.3)	15 (68.2)

*^a^* Data are reported as No. of patients (%). ISp, infraspinatus; LHB, long head of the biceps; SSc, subscapularis; SSp, supraspinatus.

### Diagnostic Performance and Observer Agreement

The results of the image assessments are summarized according to anatomic area in Table 2, and the kappa statistics for interobserver and intraobserver agreement are shown in Table 3.

**Table 2:** Diagnostic Performance Statistics by Anatomic Position*^a^*

Procedure	Sensitivity	Specificity	PPV	NPV
Rotator Cuff				
Mi-eye2				
Reviewer 1	0.75	1.00	1.00	0.77
Reviewer 2	0.50	1.00	1.00	0.63
Arthroscopy				
Reviewer 1	0.67	0.90	0.89	0.69
Reviewer 2	0.42	0.80	0.71	0.53
MRI	0.75	0.75	0.82	0.67
Long Head of the Biceps				
Mi-eye2				
Reviewer 1	0.67	0.95	0.67	0.95
Reviewer 2	0.33	0.90	0.33	0.90
Arthroscopy				
Reviewer 1	1.00	0.68	0.33	1.00
Reviewer 2	0.00	0.90	0.00	0.85
MRI	0.00	0.83	0.00	0.83
Labrum				
Mi-eye2				
Reviewer 1	0.33	0.50	0.45	0.39
Reviewer 2	0.42	0.70	0.63	0.50
Arthroscopy				
Reviewer 1	0.50	0.00	0.55	0.46
Reviewer 2	0.50	0.70	0.67	0.54
MRI	0.54	0.75	0.78	0.88
Cartilage				
Mi-eye2				
Reviewer 1	0.00	0.94	0.00	0.81
Reviewer 2	0.50	0.89	0.50	0.89
Arthroscopy				
Reviewer 1	0.50	0.83	0.40	0.88
Reviewer 2	0.25	0.72	0.17	0.81
MRI	0.50	0.94	0.67	0.88
Rotator Interval				
Mi-eye2				
Reviewer 1	0.73	0.64	0.67	0.70
Reviewer 2	0.09	1.00	1.00	0.52
Arthroscopy*^b^*				
Reviewer 1	0.82	0.64	0.69	0.78
MRI	0.10	0.90	0.50	0.50

*^a^* MRI, magnetic resonance imaging; NPV, negative predictive value; PPV, positive predictive value.

*^b^* The arthroscopic image data were not able to be analyzed for reviewer 2.

**Table 3:** Kappa Statistics for Intraobserver and Interobserver Agreement

Location and Reader	Intraobserver κ (Mi-eye2 vs Arthroscopy)	Interobserver κ (Mi-eye2, Reviewer 1 vs 2)
Rotator cuff		0.703
Reviewer 1	0.624	
Reviewer 2	0.455	
Long head of the biceps		0.228
Reviewer 1	0.371	
Reviewer 2	0.327	
Labrum		0.522
Reviewer 1	0.091	
Reviewer 2	–0.024	
Cartilage		0.353
Reviewer 1	–0.082	
Reviewer 2	–0.023	
Rotator interval*^a^*		0.076
Reviewer 1	0.538	

*^a^* The arthroscopic image data were not able to be analyzed for reviewer 2.

#### Rotator Cuff

Twelve of the patients had a rotator cuff tear diagnosed by formal arthroscopy. Of these, 5 were partial-thickness articular-sided tears. In the identification of cuff pathology, needle arthroscopy had a sensitivity of 0.75, specificity of 1.00, PPV of 1.00, and NPV of 0.77 for reviewer 1 and a sensitivity of 0.50, specificity of 1.00, PPV of 1.00, and NPV of 0.63 for reviewer 2. MRI exhibited a sensitivity of 0.75, specificity of 0.75, PPV of 0.82, and NPV of 0.67 (Table 2). Substantial interobserver agreement was found regarding the Mi-eye2 images (κ = 0.703). The intraobserver agreement between the Mi-eye2 and arthroscopy images was substantial for reviewer 1 and moderate for reviewer 2 (κ = 0.624 and 0.455, respectively) (Table 3).

#### Long Head of the Biceps

Regarding the identification of long head of the biceps pathology, needle arthroscopy had a sensitivity of 0.67, specificity of 0.95, PPV of 0.67, and NPV of 0.95 for reviewer 1; a sensitivity of 0.33, specificity of 0.90, PPV of 0.33, and NPV of 0.90 for reviewer 2; and a sensitivity of 0.00, specificity of 0.83, PPV of 0.00, and NPV of 0.83 for MRI (Table 2). The interobserver agreement on the Mi-eye2 images was fair (κ = 0.228), and the intraobserver agreement between the Mi-eye2 and arthroscopy images was fair for both reviewers (κ = 0.371 for reviewer 1 and 0.327 for reviewer 2) (Table 3).

#### Labrum

Twelve of the included patients had labral pathology, identified by the primary surgeon on formal arthroscopy. Eight involved the anteroinferior labrum and 4 involved the posterior labrum. In the identification of labral pathology, needle arthroscopy had a sensitivity of 0.33, specificity of 0.50, PPV of 0.45, and NPV of 0.39 for reviewer 1; a sensitivity of 0.42, specificity of 0.70, PPV of 0.63, and NPV of 0.50 for reviewer 2; and a sensitivity of 0.54, specificity of 0.75, PPV of 0.78, and NPV of 0.88 for MRI (Table 2). Moderate interobserver agreement was found regarding the Mi-eye2 images (κ = 0.522). The intraobserver agreement between the Mi-eye2 and arthroscopy images was none to slight for reviewer 1 and reviewer 2 (κ = 0.091 and –0.024, respectively) (Table 3).

#### Cartilage

In the identification of cartilage pathology (of either the humeral head or glenoid), needle arthroscopy had a sensitivity of 0.50, specificity of 0.83, PPV of 0.40, and NPV of 0.88 for reviewer 1; a sensitivity of 0.25, specificity of 0.72, PPV of 0.17, NPV of 0.81 for reviewer 2; and a sensitivity of 0.50, specificity of 0.94, PPV of 0.67, and NPV of 0.88 for MRI (Table 2). The interobserver agreement for the Mi-eye2 images was fair (κ = 0.353), and the intraobserver agreement between the Mi-eye2 and formal arthroscopy images was none to slight for both reviewers (κ = –0.082 for reviewer 1 and –0.023 for reviewer 2) (Table 3).

#### Rotator Interval

Eleven of the patients had rotator interval pathology. Needle arthroscopy for identifying rotator interval pathology had a sensitivity of 0.73, specificity of 0.64, PPV of 0.67, and NPV of 0.70 for reviewer 1; a sensitivity of 0.09, specificity of 1.00, PPV of 1.00, and NPV of 0.52 for reviewer 2; and a sensitivity of 0.10, specificity of 0.90, PPV of 0.50, and NPV of 0.50 for MRI (Table 2). None/to slight interobserver agreement was found for the Mi-eye2 (κ = 0.076). The intraobserver agreement between the Mi-eye2 and formal arthroscopy images was moderate for reviewer 1 (κ = 0.538) (Table 3). The formal arthroscopic image data were unable to be analyzed for reviewer 2; thus, it was not possible to calculate an intraobserver statistic for this reviewer.

## Discussion

Our study was performed to assess the diagnostic accuracy of a needle arthroscopy device in the glenohumeral joint. The device performed favorably when compared to MRI in the diagnosis of rotator cuff and long head of the biceps pathology, with comparable results for the rotator interval. However, it did not perform as well in the diagnosis of labral and cartilaginous pathology.

In the assessment of a patient with shoulder pain, commonly used diagnostic modalities include MRI and ultrasonography. Ultrasonography is cheap, noninvasive, and readily available, with a high diagnostic accuracy for full-thickness rotator cuff tears.^7^ However, it is operator dependent, less accurate for partial-thickness cuff tears, and relatively unproven in labral assessment.^7,10^ MRI is of high accuracy for numerous intra-articular pathologies, although it is often of limited access and contraindicated in some patients.^6^ Thus, needle arthroscopy represents an alternative modality, for use at the initial point of contact.

For the rotator cuff, this study has found greater accuracy of the needle arthroscope to “rule in” tears than MRI, as reflected by high specificities and PPVs. This is in concordance with a recent study of the modality.^11^ However, in contrast to that paper, this study found similar sensitivities and NPVs, indicating that needle arthroscopy is comparable to MRI at “ruling out” cuff tears. This likely reflects the limitations of MRI in the detection of partial-thickness cuff tears, with reported sensitivities in the literature ranging from 0.52 to 0.91.^1,8^

Our study also found needle arthroscopy to have greater accuracy than MRI in both “ruling in” (higher specificities and PPVs) and “ruling out” (higher sensitivities and NPVs) long head of the biceps pathology. MRI has previously been demonstrated to be of high accuracy for full-thickness biceps tendon tears, with a diminished accuracy for partial-thickness lesions, inflammation, and fraying.^3,4^ The direct tendon visualization of needle arthroscopy thus provides a greater accuracy for these lesions.

The needle arthroscopy device performed less accurately than MRI in all measures for labral pathology and in sensitivities and PPVs for cartilaginous pathology. This is likely because of the visualization limitations of the device, observed here with low overall adequate visualization percentages of the posterior labrum (18.2% and 22.7%) and glenoid (77.3%) by the blinded reviewers. Indeed, this device consists of a 0° arthroscope, offering a reduced total field compared with a conventional 30° arthroscope. Furthermore, as a single-portal diagnostic device, it does not provide capability to probe structures (eg, to assess cartilaginous consistency). Assessment of the rotator interval was inconsistent between the reviewers, as reflected by a poor interobserver kappa score. However, here the Mi-eye2 was overall more accurate than MRI.

### Limitations

This study is subject to a number of limitations. Twenty-two patients is a relatively small sample number, confounded by the varying prevalence of different pathologies (although the majority had rotator cuff or labral pathology). Assessment of static photographic images, as performed by our reviewers, is inferior to real-time dynamic assessment. This study did not include assessment of the subacromial bursa. It should be noted that this study did not assess the safety and feasibility in the outpatient setting, as procedures were performed under anesthesia in a sterile surgical field, before formal arthroscopy. Further work should assess patient satisfaction, pain scores, and complications (including systemic complications, such as syncope) for procedures performed under local anesthesia.

## Conclusion

In this study, we found needle arthroscopy to be more accurate than MRI in the assessment of rotator cuff and long head of the biceps pathology, although less accurate for the articular cartilage and labrum. It may offer a suitable alternative to advanced imaging for these conditions in the outpatient setting or where MRI is inconclusive. Further work is required to assess real-world applicability in the clinic. The development of a needle arthroscope with an angled lens may further advance the potential diagnostic and therapeutic applications.

## References

[1] Brockmeyer M Schmitt C Haupert A Kohn D Lorbach O . Limited diagnostic accuracy of magnetic resonance imaging and clinical tests for detecting partial-thickness tears of the rotator cuff. Arch Orthop Trauma Surg. 2017;137(12):1719–1724. 28942510 10.1007/s00402-017-2799-3

[2] Gill TJ Safran M Mandelbaum B Huber B Gambardella R Xerogeanes J . A prospective, blinded, multicenter clinical trial to compare the efficacy, accuracy, and safety of in-office diagnostic arthroscopy with magnetic resonance imaging and surgical diagnostic arthroscopy. Arthroscopy. 2018;34(8):2429–2435. 29804955 10.1016/j.arthro.2018.03.010

[3] Kim JY Rhee SM Rhee YG . Accuracy of MRI in diagnosing intra-articular pathology of the long head of the biceps tendon: results with a large cohort of patients. BMC Musculoskelet Disord. 2019;20(1):1–9. 31153372 10.1186/s12891-019-2654-5 PMC6545217

[4] Lee RW Choi SJ Lee MH , et al. Diagnostic accuracy of 3 T conventional shoulder MRI in the detection of the long head of the biceps tendon tears associated with rotator cuff tendon tears. Skeletal Radiol. 2016;45(12):1705–1715. 27717975 10.1007/s00256-016-2501-9

[5] Liu F Cheng X Dong J Zhou D Han S Yang Y . Comparison of MRI and MRA for the diagnosis of rotator cuff tears: a meta-analysis. Medicine (Baltimore). 2020;99(12):e19579. 32195972 10.1097/MD.0000000000019579 PMC7220562

[6] Liu F Cheng X Dong J , et al. Imaging modality for measuring the presence and extent of the labral lesions of the shoulder: a systematic review and meta-analysis. BMC Musculoskelet Disord. 2019;20(1):487. 31656171 10.1186/s12891-019-2876-6 PMC6815459

[7] Roy JS Braën C Leblond J , et al. Diagnostic accuracy of ultrasonography, MRI and MR arthrography in the characterisation of rotator cuff disorders: a systematic review and meta-analysis. Br J Sports Med*.* 2015;49(20):1316–1328. 25677796 10.1136/bjsports-2014-094148 PMC4621376

[8] Sharma G Bhandary S Khandige G Kabra U . MR imaging of rotator cuff tears: correlation with arthroscopy. J Clin Diagn Res. 2017;11(5):TC24–TC27. 28658874 10.7860/JCDR/2017/27714.9911 PMC5483776

[9] Shin JJ Popchak AJ Musahl V Irrgang JJ Lin A . Complications after arthroscopic shoulder surgery: a review of the American Board of Orthopaedic Surgery database. J Am Acad Orthop Surg Glob Res Rev. 2018;2(12):e093. 30680371 10.5435/JAAOSGlobal-D-18-00093 PMC6336573

[10] Tat J Tat J Theodoropoulos J . Clinical applications of ultrasonography in the shoulder for the orthopedic surgeon: a systematic review. Orthop Traumatol Surg Res. 2020;106(6):1141–1151. 32763009 10.1016/j.otsr.2020.06.005

[11] Wagner ER Woodmass JM Zimmer ZR , et al. Needle diagnostic arthroscopy and magnetic resonance imaging of the shoulder have comparable accuracy with surgical arthroscopy: a prospective clinical trial. Arthroscopy. 2021;37(7):2090–2098. 33798653 10.1016/j.arthro.2021.03.006

[12] Zhang K Crum RJ Samuelsson K Cadet E Ayeni OR . In-office needle arthroscopy: a systematic review of indications and clinical utility. Arthroscopy. 2019;35(9):2709–2721. 31416656 10.1016/j.arthro.2019.03.045

